# Sterilized human skin graft with a dose of 25 kGy provides a privileged immune and collagen microenvironment in the adhesion of Nude mice wounds

**DOI:** 10.1371/journal.pone.0262532

**Published:** 2022-01-27

**Authors:** Jurandir Tomaz de Miranda, Fabiana de Andrade Bringel, Ana Paula Pereira Velosa, Verônica Protocevich, Sandra de Morais Fernezlian, Pedro Leme Silva, Vera Luiza Capelozzi, Monica Beatriz Mathor, Walcy Rosolia Teodoro

**Affiliations:** 1 Rheumatology Division of the Hospital das Clinicas da Faculdade de Medicina da Universidade de São Paulo, FMUSP, São Paulo, SP, Brazil; 2 Center of Radiation Technology (CTR) Institute of Energy and Nuclear Research, IPEN—CNEN/SP, São Paulo, Brazil; 3 Department of Pathology, Hospital das Clínicas, HCFMUSP, São Paulo, Brazil; 4 Laboratory of Pulmonary Investigation, Institute of Biophysics Carlos Chagas Filho, Health Sciences Center, Federal University of Rio de Janeiro, Rio de Janeiro, Brazil; 5 National Institute of Science and Technology for Regenerative Medicine, Rio de Janeiro, Brazil; Universidade Federal Fluminense, BRAZIL

## Abstract

This study aimed to report the effects of different doses of ionizing radiation on inflammatory and repair stage of human skin graft adherence in Nude mice wounds. Animals were divided into transplanted with irradiated human skin grafts (IHSG) at 25 and 50 kGy (IHSG 25 kGy; IHSG 50 kGy) and non-IHSG and euthanized on the 3rd, 7th and 21st days after the surgery, by gross and microscopic changes, immunostaining for human type I collagen (Col I) and mouse Col I and Col III and inflammatory cells. We found an effectiveness of human split-thickness graft adherence in mice transplanted with IHSG 25 kGy, as well decrease in dermo-epidermal necrosis and neutrophils, lower loss of skin thickness, epithelization and neo-vascularization. Day 21 post-transplantation with IHSG 25 kGy was observed a well-preserved human skin in the border of the graft, a prominent granulation tissue in an organization by proliferated fibroblasts, Col III deposition and increased B-cells and macrophages. A complete adherence of human skin graft occurred with IHSG 25 kGy. We suggest that the ionizing radiation at 25 kGy mediates inflammation and the repair stage of human skin graft adherence in murine model, thus emerging as a potential tool in healing cutaneous wounds.

## Introduction

The injuries caused by skin burns with high extension are an important problem in public health. These injuries result in a high mortality and morbidity rate due to the extensive contractures, restriction of movement, itchiness and pain, dissatisfaction with appearance and loss of function for long periods of time [1–3].

The epidermis is the superficial layer of the skin comprised by stratifying layers of keratinocytes, thus working as the primary barrier and biosensor to the external environment. The dermis is beneath of the epidermis, to provide the strength of cytoskeleton to the skin. It is primarily composed of type I collagen (Col I) produced by dermal fibroblasts. The skin normal healing process involves the inflammatory, proliferative and tissue remodeling phases. In the inflammatory phase, initially there is an increased influx of neutrophils followed by macrophages, which release a series of cytokines, essential for the removal of devitalized tissue. The proliferative phase involves the proliferate and migration of fibroblasts from the uninjured areas to the wound region [4]. At this stage, fibroblasts mainly secrete type III collagen, forming granulation tissue. Furthermore, fibroblasts can differentiate into myofibroblasts, which have the contractile capacity to reach the wound edges, besides neoangiogenesis results in the vascularization of the dermis and the proliferation of keratinocytes favors the re-epithelialization and closure of the epidermis [5–7]. The remodeling phase is characterized by extracellular matrix remodeling, replacing scar tissue with a physiological matrix, constituted mainly of Col I [6,8,9].

Recently, several non-dermic matrixes have been proposed to replace human skin grafts, including synthetic, semisynthetic or natural products [10,11]. However, despite the advances in tissue bioengineering, skin allografts continue to serve as the “gold standard” for the temporary coverage of extensive and deep burns [12]. The allografts have several desirable properties since they present the capacity to adhere the wound course, providing a better closure, promoting the retention of humidity while improving the thermoregulation of the grafted location [13–16].

However, one concern about the graft during the tissue storage process is the contamination. For this reason, the agency of International Atomic Energy (IAEA) is promoting and encouraging the development of international and national tissue database programs, with the goal of using ionizing radiation as a final sterilization in human tissues for transplants [17,18]. The efficacy of the radiation results from its high capacity to penetrate the matter and its high efficiency in inactivating microorganisms, which allows for the sterilization of materials that are already in sealed packages and prevents posterior recontamination. It is well-known that doses above 25 kGy can lead to alterations of the dermis, promoting significant alterations of the Col I fibers [19,20], with biomechanical properties alterations. Nevertheless, it is not known the effects of different doses of ionizing radiation on inflammatory and remodeling stage of human skin graft adherence, which may occur before biomechanical properties can be detected.

Considering the growing need for tissue biosafety and bioengineering of biomaterials in reconstructive plastic surgery, the possibility of expanding the scope of radio-sterilized grafts represents a major challenge. This study aims to report the effects of different doses of ionizing radiation on the inflammatory stages, matrix remodeling and adherence of human skin grafts in an allograft model in Nude mice.

## Materials and methods

### Ethics statement

All experiments with animals were conducted in accordance with the policies and guidelines for the use and care of animals of the University of São Paulo—USP and the Institute of Energy and Nuclear Research—IPEN, and approved by the Ethics Committee in Animal Research (Protocol no. 14/CEPA-IPEN/SP) and the Ethics Committee for Research in Human Beings (Protocol No. 772/ CEP) of the Institute of Biomedical Sciences, University of São Paulo, approved this study.

### Mice

Forty-two male and female NUDE mice (Nu/Nu), 8 weeks of age, from Bioterium of the Energy Research Institute of the National Nuclear Energy Commission, IPEN–CNEN/SP were maintained at 23°C under a 12 h light-dark cycle with access to food and water ad libitum in the vivarium of the Center for Biotechnology of the Institute of Energy and Nuclear Research—IPEN. The mice were monitored by a responsible veterinarian daily, from the beginning to the end of the study in animals.

### Human skin graft in Nude mice

Human skin from multi-organ donors was obtained from the Tissue Bank of the Central Institute of Clinics of the University of São Paulo School of Medicine Hospital (Protocol number 2.600). The human skin samples are 0.40 to 0.60 mm thick, these samples were cut to a size of 2.0 cm2 and then soaked in glycerol and wrapped in polyethylene (Whirl-Pak® Write-On Bag, Nasco, Fort Atkinson, Wisconsin, USA), and submitted in Cobalt60 irradiator (Gammacell-220, Nordion Inc., Quebec, CA), under the following conditions: 2°C to 8°C, 3,484.292 Ci activity and 2.88 kGy/h dose rate. Human skin after being treated and sterilized by radiation was used as a graft in NUDE mice.

For the surgical procedure, Nude mice were anaesthetized by intramuscular injection of 5% ketamine (Ventanarcol, Koing do Brasil Ltda., São Paulo, BR) and xylazine 2% (Kensol®, Konig do Brasil Ltda., São Paulo, BR) with a physiological solution. These mice were randomly divided into 3 groups: grafted with human skin irradiated with a dose of 25 kGy (IHSG 25 kGy; n = 14), 50 kGy (IHSG 50 kGy; n = 14) and non-irradiated (control group non-IHSG; n = 14) (S1 Fig). These IHSG and non-IHSG were transplanted onto a 1.5 x 1.5 cm2 wound created with a scalpel on the dorsal skin of the animals and maintained in a surgical titanium alloy utilizing clips LT100 (Ethicon Endo-Surgery Inc., Cincinnati, Ohio, USA) and fibrin glue (TissueBond®, Recife, PE, BRA). For postoperative analgesia, meloxicam-0,2mg/Kg (IM) and Buprenorphine-0,05mg/Kg (SC) was provided for 3 days.

The mice were observed daily by the responsible veterinarian of the Center for Biotechnology, they were treated and cared for according to the international standards of animal care.

Euthanasia took place at 3, 7, 21 and 90 days after grafting, and followed the following patterns: A dose of 2% xylazine hydrochloride (kensol, koing do Brasil Ltda, São Paulo, BR) and 5% ketamine hydrochloride (Vetanacol, Kong do Brasil Ltda, São Paulo, BR) were allocated in a carbon dioxide chamber (Beira Mar Model GSCO2, São Paulo, BR).

Soon after euthanasia, the skin of the mice’s dorsal region, where the graft was located, was removed for histological procedures.

### Gross examination

At the macroscopic level, the behavior of the graft was evaluated from 3 to 21 days in the IHSG 25 kGy, IHSG 50 kGy and non-IHSG transplanted in mice. A group was maintained by 90 days to evaluate the gross tissue contraction and presence of hypertrophic scarring.

### Microscopic examination

Tissue fragments passing from the border to the center plane of each wound were 10% paraformaldehyde-PBS-fixed for 24h, embedded in paraffin and 4μm slices were stained with hematoxylin-eosin (H&E). All of the histological analyses were performed in a blinded manner by pathologist (VLC) to determine the distribution of dermo-epidermal necrosis, neutrophils, skin thickness, keratinocytes epithelization, small vessels, macrophages, and granulation tissue.

### Immunohistochemistry

To identify inflammatory cells (T-cell and B-cell lymphocytes) and macrophages. The slides were dewaxed in xylene and rehydrated. For the recovery of the antigenic sites, the slides were placed at high temperature in citrate solution (pH 6) for 1 minute at 125°C. The endogenous peroxidase blockade was made with hydrogen peroxide 10% and methanol 3% (v/v) and, by immersing in 2 baths for 10 minutes each. the nonspecific sites were blocked with BSA (Bovine Serum Albumin, Sigma-Aldrich, St. Louis, MO, USA) at 5% for 20 minutes at room temperature. The slides were incubated overnight at 4°C with the following mouse monoclonal antibodies diluted in 1% BSA: anti-human CD4 (1:100, Biorbyt, Cambridge, UK), CD20 (1:150, Biorbyt, Cambridge, UK) and a CD68 antibodies (1:500, Biorbyt, Cambridge, UK). For negative controls, the sections were incubated with 1% BSA. The reaction was revelated with the Mouse-PK 6101 kit (ABCElite Kit Vectastain-Vector Lab California, USA), the Diaminobenzidine (DAB, Sigma-Aldrich Chemie, Steinheim, DE) was used as the chromogen. Subsequently, the slides were counterstaining with Harris Hematoxylin (Merck, Darmstadt, DE).

### Immunofluorescence

To identify human Col I and mouse Col I and III fibers, skin sections were dewaxed in xylene, rehydrated and subjected to pepsin (Sigma Chemical Co., St. Louis, MO, USA) (8 mg/ml) in acetic acid 0.5 N, for 30 minutes at 37°C. Nonspecific sites were blocked with 5% BSA for 30 minutes at room temperature. The slides were incubated overnight at 4°C with rabbit anti-human Col I (1:50; Rockland, Gilbertsville, PA, USA), rabbit anti-mouse Col I (1:40; Abcam, Cambridge, UK) and rabbit anti-mouse Col III antibodies (1:200; LSBio, Seattle, WA, USA) diluted in PBS. Negative controls were incubated with PBS. After washings with PBS-0.05% Tween20, skin sections were incubated for 60 minutes with goat anti-rabbit Alexa Fluor 488 (Thermo Fisher Scientifc, Rockford, IL, USA) diluted 1:200 in PBS solution, containing 0.006% Evans blue (Sigma-Aldrich, St. Louis, MO, USA). The sections were mounted with a buffered glycerol solution and examined under a fluorescence microscope (Olympus BX-51, Olympus Co., Tokyo, JP).

### Histomorphometry

Histologic parameters, B-cells lymphocytes and macrophages CD68+, and collagen fibers immunofluorescence were evaluated by histomorphometry (Image Pro-Plus 6.0, Rockville, MD, USA) in six different, randomly selected fields including the borders and center of the skin. At 400x magnification, the number of positive cells in each field was calculated according to the number of points hitting positive cells as a proportion of the total grid area.

Collagenous fibers (human Col I, and mouse Col I and III) were quantified by optical density at the image analysis system (Image Pro-Plus 6.0, Rockville, MD, USA) in 6 different and randomly selected. The results were expressed as the percentage of collagen fibers/dermis collagen tissue.

The results were expressed as percentage of positive cells/epidermis or dermis connective tissue. All measurements were taken by 2 independent researchers (JTM and WRT) who were blinded to the treatment group.

### Statistical analysis

The Kolmogorov–Smirnov test with Lilliefors correction was used to assess normality of data, while the Levene median to evaluate the homogeneity of variances. The data were expressed as the mean ± standard deviation or median and interquartile range, according to data distribution. Data were compared by student t-test or one-way ANOVA with Dunnett post hoc tests for multiple comparisons for normal data distribution and homogeneous variances. The GraphPad Prism 5.0 software (San Diego, CA, USA) was used and p value P<0.05 was considered statistically significant.

## Results

**Fig 1:**
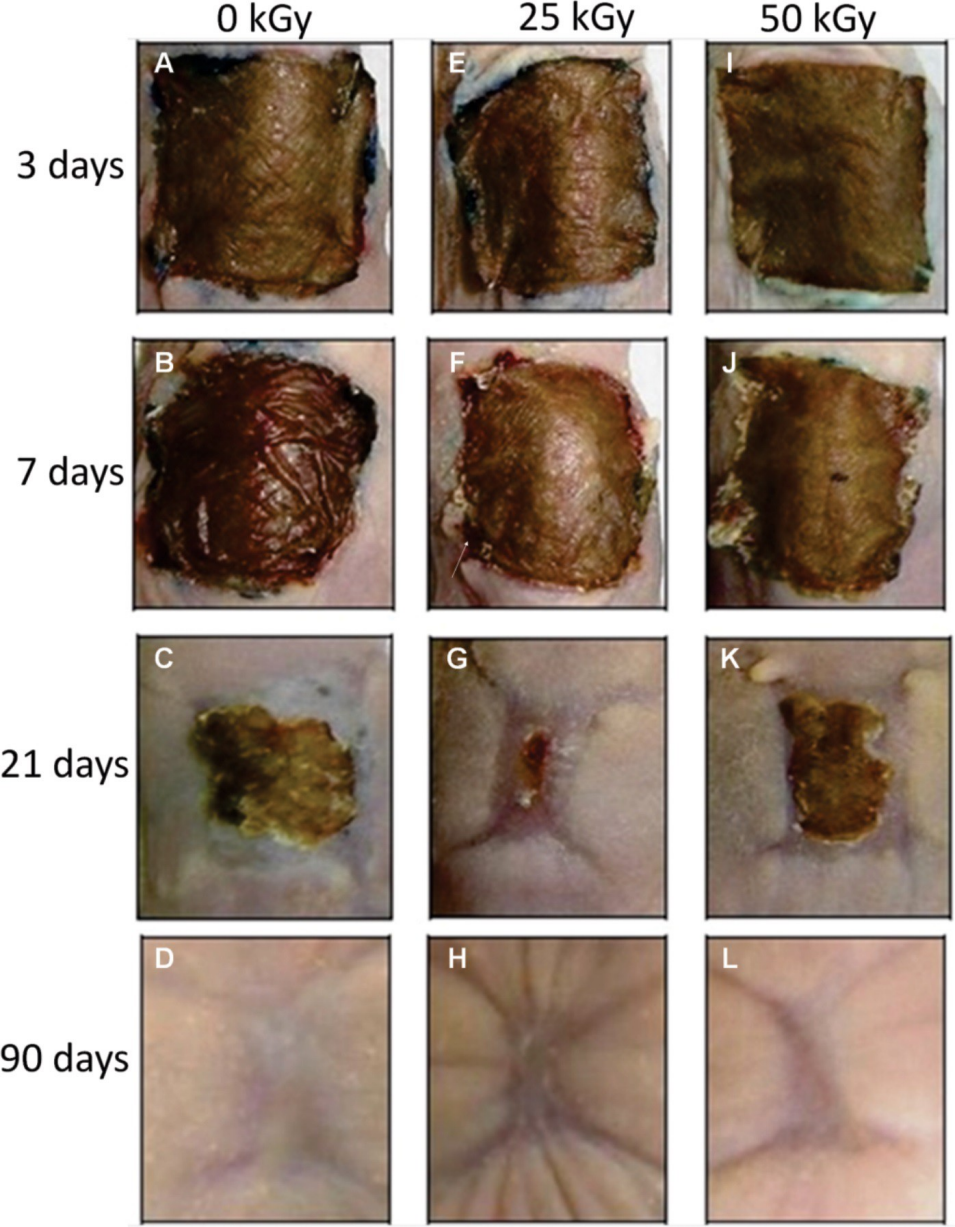
Macroscopic evolution of the human skin graft in Nude mouse over time. Human skin graft control (non-irradiation), group 3 (A), 7 (B), 21 (C) and 90 (D) days after surgery, (B) tissue was occupied by an extensive area of necrosis and haemorrhage. Human skin graft with dose 25 kGy group 3 (E), 7 (F), 21 (G) and 90 (H), in (G) only small central crusted island observed, (H) greater tissue contraction. Human skin graft with dose 50 kGy group 3 (I), 7 (J), 21 (K) and 90 (L), the graft was retracted and pale (J) the border was occupied by an extensive area of necrosis and haemorrhage, (K) central crusted island remains. All grafts show complete regeneration in 90 days (D), (H), (L).

Fig 1 shows a gross examination of the animal model and course of wound healing after non-irradiated and irradiated human skin graft over time. After the first 3 days, non-irradiated and irradiated stable human IHSGs in mice take over effectively. Macroscopically, these grafts were soft and pliable after 3 days, resembling normal human skin (Fig 1A, 1E and 1I). The IHSGs featured normal coloration in all animals during days 3 and 7 (Fig 1) but successively covered the crusty skin formation over the first 21 days, mainly in animals transplanted with IHSG 25 kGy (Fig 1E–1G). The compressive effects of grafts produced ischemia and resulted in bleeding during the first 7 days post-transplantation (Fig 1). Then, a further evolution towards a retracted and elevated lesion with crusty edges and underlying granulation tissue was observed in all animals. The appearance of the lesion changed completely on day 21 due to the border loss of the crust and the formation of a sclerotic surface (Fig 1C, 1G and 1K). The crust progressively disappeared and was replaced by a fully re-epithelialized area resembling normal and fine human skin. The effect of the contraction and retraction of the grafted skin was maximum for IHSG 25 kGy (Fig 1G) compared to non-IHSG and IHSG 50 kGy in the first 21 days (Fig 1C and 1K). The (Fig 1D, 1H and 1L) shows the healing aspect 90 days after of the human graft in the non-IHSG, IHSG 25 kGy and IHSG 50 kGy groups.

**Fig 2:**
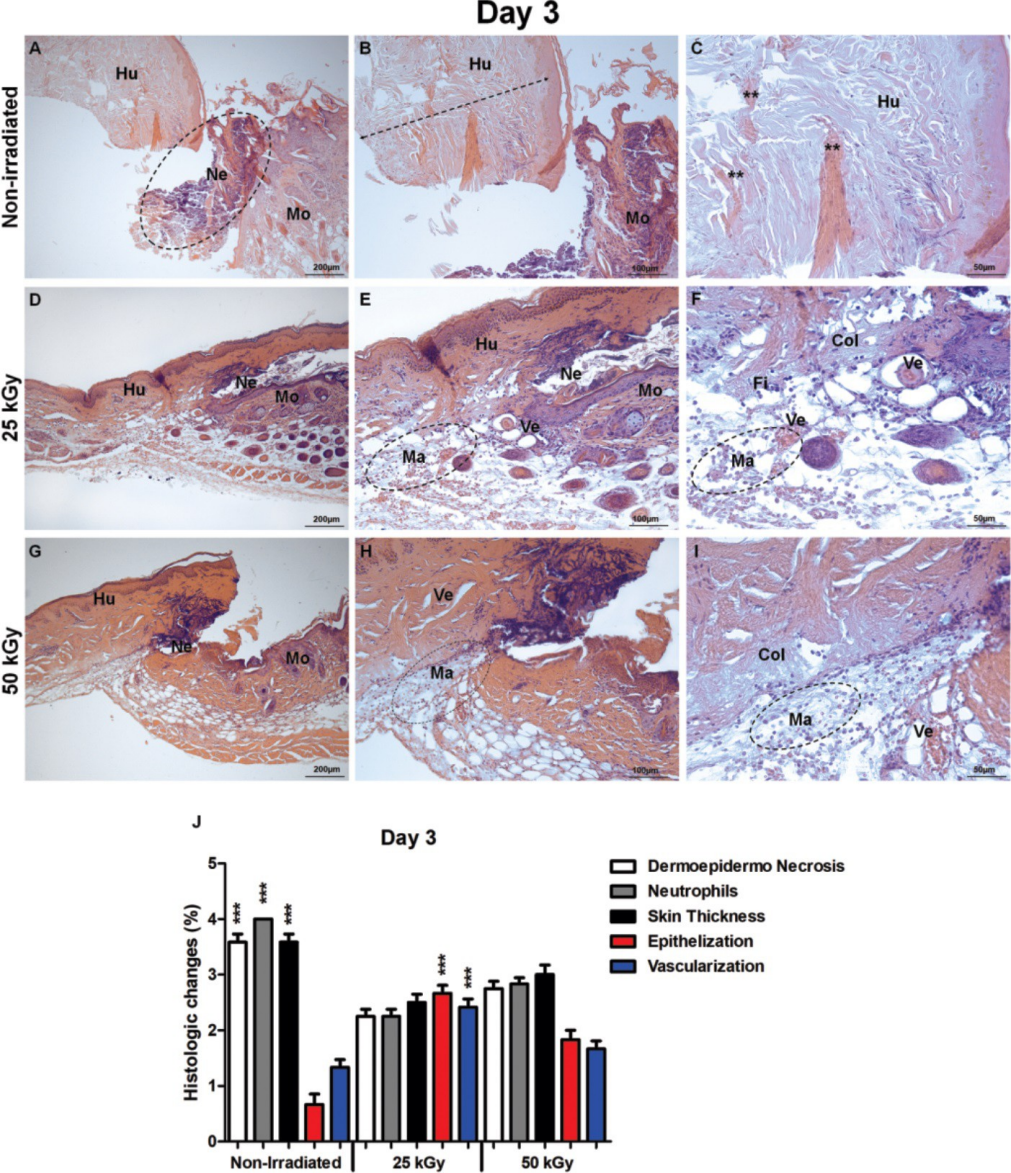
Histological analysis of irradiated and non-irradiated human skin grafts, 3 days after grafting. (A-C) non-irradiated group. Human skin graft (Hu), mouse skin (Mo). Dermoepidermal necrosis, neutrophil exudate (Ne, A). Total graft thickness (dotted arrow, B). Human skin collagen (**, C). (D-F) group irradiated 25 kGy. Dermoepidermal necrosis, neutrophil exudate (Ne, D, E), reepithelialization, macrophage infiltration (Ma, E, F), collagen, fibroblasts, blood vessels (Col, FI, Ve, E-F), of the animal. (G-I) group irradiated 50 kGy. Dermoepidermal necrosis (Ne, G), macrophage infiltration (Ma, H, I), collagen degradation (Col I). (J) statistical differences between inflammation and repair process in mice grafted with IHSG 25 kGy. Dermoepidermal necrosis, neutrophils, graft thickness decreased compared with non-irradiated group. Epithelialization, vascularization at 25 kGy increased compared to other groups. (***p<0.0001). Original magnification: 100X (A, D, G); 200X (B, E, H); 400X (C, F, I).

**Fig 3:**
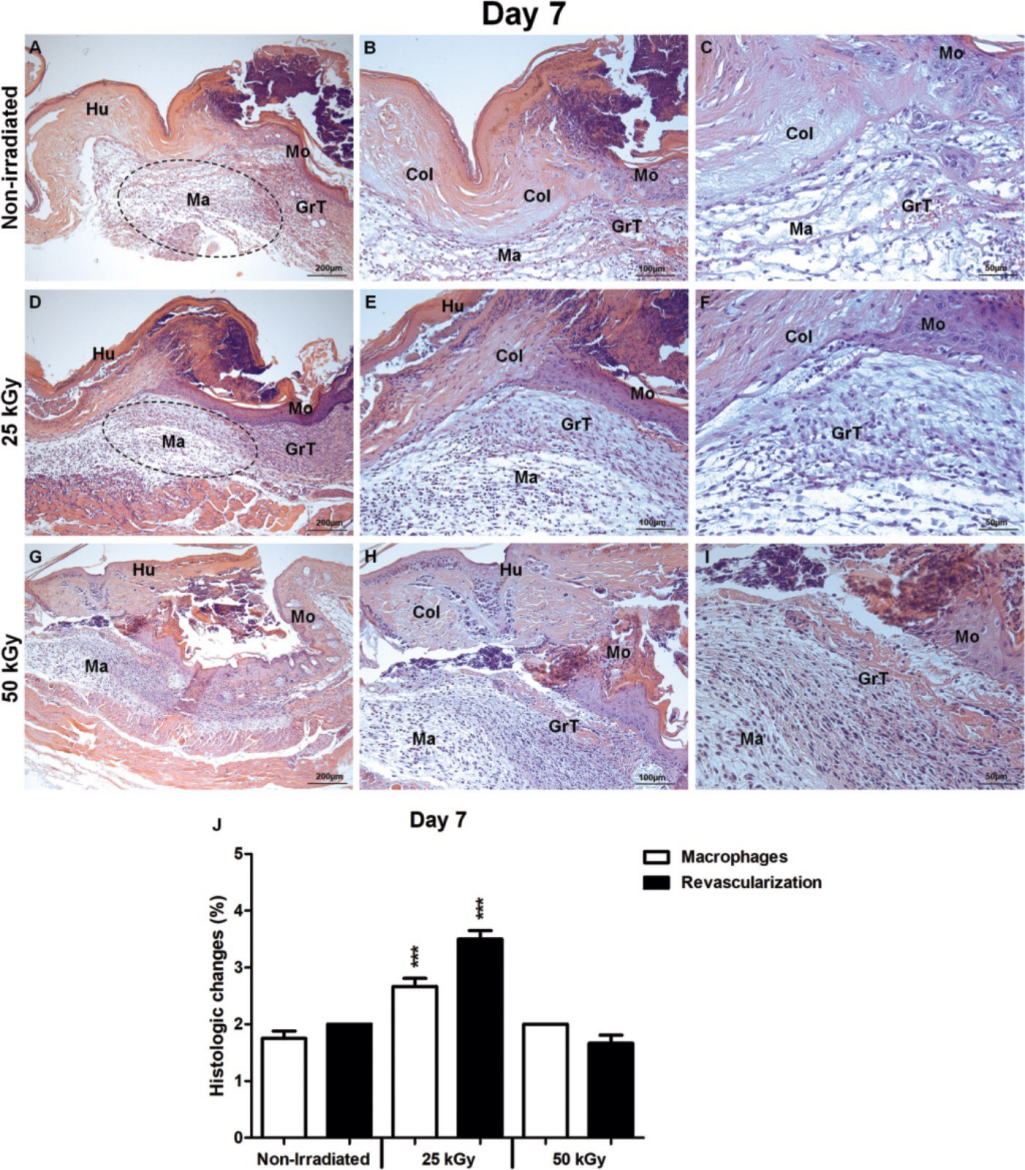
Histological analysis of irradiated and non-irradiated human skin grafts, 7 days after grafting. (A-C) non-irradiated group. Human skin graft (Hu), mouse skin (Mo), granulation tissue filling (GrT), macrophage infiltration (Ma), collagen (Col). (D-F) group irradiated 25 kGy. Macrophage infiltration (Ma), filling of granulation tissue (GrT) that progresses from the mouse dermis towards the 25 kGy IHSG graft compared to other groups. Reepithelization and collagen deposition (Col). (G-I) group irradiated 50 kGy. filling of granulation tissue (GrT), infiltration of macrophages (Ma), collagen (Col). (J) statistical differences between the process of epithelial repair in the matrix with increased macrophages and revascularization in skin wounds of mice transplanted with IHSG 25 kGy (***p<0.0001). Original magnification: 100X (A, D, G); 200X (B, E, H); 400X (C, F, I).

**Fig 4:**
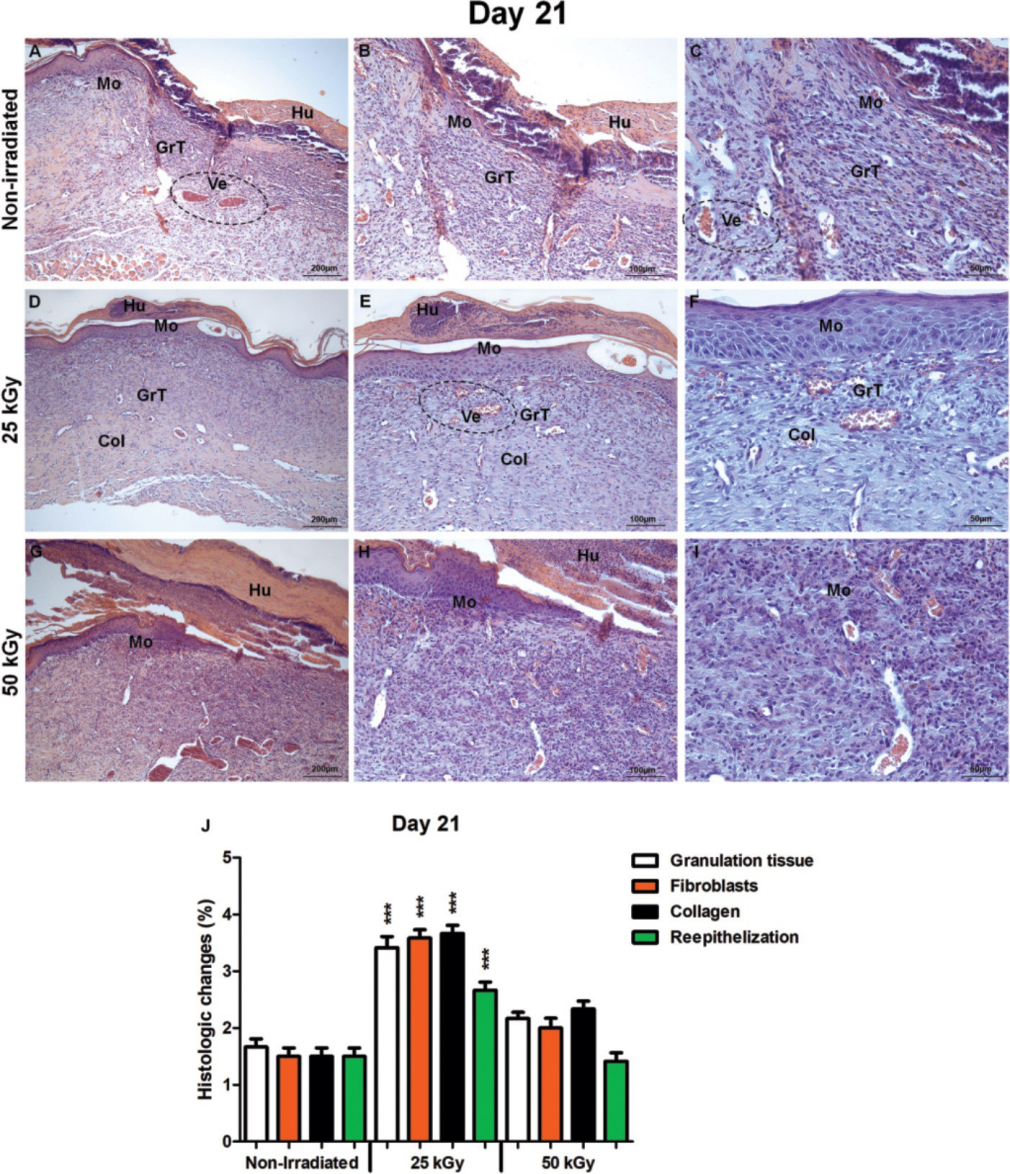
Histological analysis of irradiated and non-irradiated human skin grafts, 21 days after grafting. (A-C) non-irradiated group. Human skin graft (Hu), mouse skin (Mo), granulation tissue filling (GrT), blood vessels (Ve). (D-F) group irradiated 25 kGy. Human skin (Hu, D) detached fragment covering the center of the healing area, large blood vessels (Ve, E-F), granulation tissue (GrT, D-F) organized by proliferated fibroblasts and deposition of fine collagen fibers (Col, E-F). (G-I) group irradiated 50 kGy. Human skin graft (Hu), mouse skin (Mo) still in the healing process. (J) Statistical differences between the reepithelialization process and granulation tissue, fibroblasts, collagen in the epithelial and matrix repair process in mice grafted with IHSG 25 kGy (***p<0.0001). Original magnification: 100X (A, D, G); 200X (B, E, H); 400X (C, F, I).

The (Figs 2–4) show respectively, the histological examination of the non-IHSG, IHSG 25 kGy, and IHSG 50 kGy on mouse wound at 3rd, 7th and 21st days after grafting. The non-IHSG at day 3, a low magnification showed the mouse skin wound (host) replaced by dermo-epidermal necrosis, neutrophils exudate in the center and full thickness skin loss by extension of the process to deep dermis (Fig 2A). At high magnification, a "U" shape was formed by dermo-epidermal collagen necrosis (Fig 2B). The edges of the tissue were also affected by significant loss of epithelialization, vascularization, macrophages infiltration and collagen degradation (Fig 2B and 2C). These edges were followed by skin areas showing denuded surface, epithelial desquamation and small surface areas covered with crust. The non-irradiated human skin graft exhibited epidermis integrity, superficial dermis composed of loose connective tissue, while the deep dermis showed homogeneous bundles of acellular collagen (Fig 2A). In contrast, the skin wound of mouse transplanted with IHSG 25 kGy showed significant mitigation of the dermo-epidermal necrosis (Fig 2D and 2J) and neutrophil exudate (Fig 2E and 2J), as well as beginning of the re-epithelization, increased macrophages infiltration, and neo-vascularization by granulation tissue and decreased collagen degradation (Fig 2F and 2J) when compared to non-IHSG (Fig 2A–2C and 2J) and IHSG 50 kGy (Fig 2G–2J) (Fig 2J; p<0.0001).

At day 7, an increased macrophage infiltrate filled an exuberant granulation tissue progressing from superficial and deep dermis of IHSG 25 kGy (donate) to the mouse wound (receptor) (Fig 3D–3F) compared to non-IHSG (Fig 3A–3C) and IHSG 50 kGy (Fig 3G–3I), being this difference statistically significant (Fig 3J; p<0.0001). In IHSG 25kGy, the granulation tissue is composed by an effective neo-vascularization which implies graft stability after this procedure (Fig 3D–3F). At this stage, the host tissue presented the usual features of mouse skin. It is worth noting that this animal model had a fine skin, two or three layers of keratinocytes, a papillary dermis with well-preserved hair follicles and a deep dermis with great development of adipose tissue.

After the first 21 days post-grafting of IHSG 25 kGy, the presence of well-preserved human skin could be observed in the center of the graft which was formed by a stratified epidermis with many papillae (Fig 4D–4F). The dermis layer exhibited well-vessels formation and the granulation tissue in the organization by proliferated fibroblasts, and deposition of fine collagen fibers (Fig 4D–4F). In contrast, the mouse wound skin remained open with no re-epithelization by the non-IHSG (Fig 4A), accompanied by poor neo-vascularization and immature granulation tissue in organization by collagen fibers (Fig 4B and 4C). A similar pattern could be observed in IHSG 50 kGy (Fig 4G–4I). These differences in repair process of the mouse wound skin were statistically significant (Fig 4J; ***p<0.0001).

**Fig 5:**
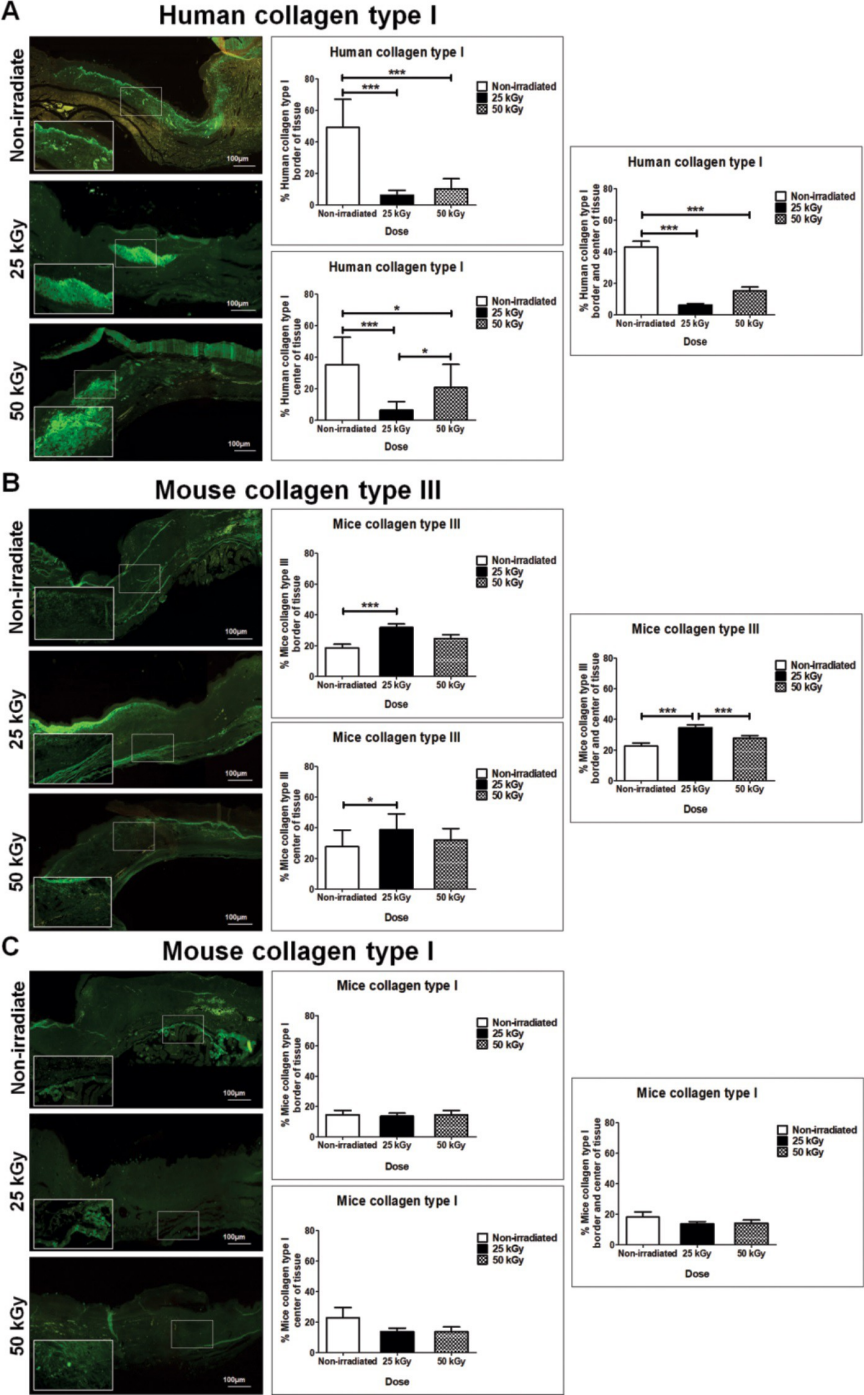
Characterization by immunofluorescence of the distribution of collagen fibers in dermis, 21 days after grafting. Human type I collagen (A) greater statistical significance in the center and border of the non-IHSG group compared to irradiated groups, this difference being more pronounced 25 kGy IHSG tissues. Mouse type III collagen (B) greater statistical significance in the center and border of the IHSG 25 kGy group compared to non-IHSG and IHSG 50kGy. Mouse type I collagen (C), similar distribution in the three groups. Graphs showing significant differences between the repair process of the collagen matrix in skin wounds of grafted mice (A, B; *p<0.01; ***p<0.0001). Original magnification: 100X (A, B, C).

The Fig 5 shows the immunofluorescence characterization of collagen fibers distribution in dermis from human Col I and mouse Col I and III on the 21st day after the skin grafting. At this stage, the IHSG 25 kGy and IHSG 50 kGy implants in mouse showed a significant decrease of green-fluorescence of human Col I fibers at the center and border regions compared to non-IHSG implants, being this difference more pronounced in the IHSG 25 kGy implant (Fig 5A; *p<0.01). In contrast, a significant increase of green-fluorescence of mouse Col III fibers, was observed at the center and border of the IHSG 25 kGy compared to non-IHSG and IHSG 50 kGy (Fig 5B; ***p<0.0001). A similar distribution of mouse Col I is present along of the dermis of the three grafting groups (Fig 5C).

**Fig 6:**
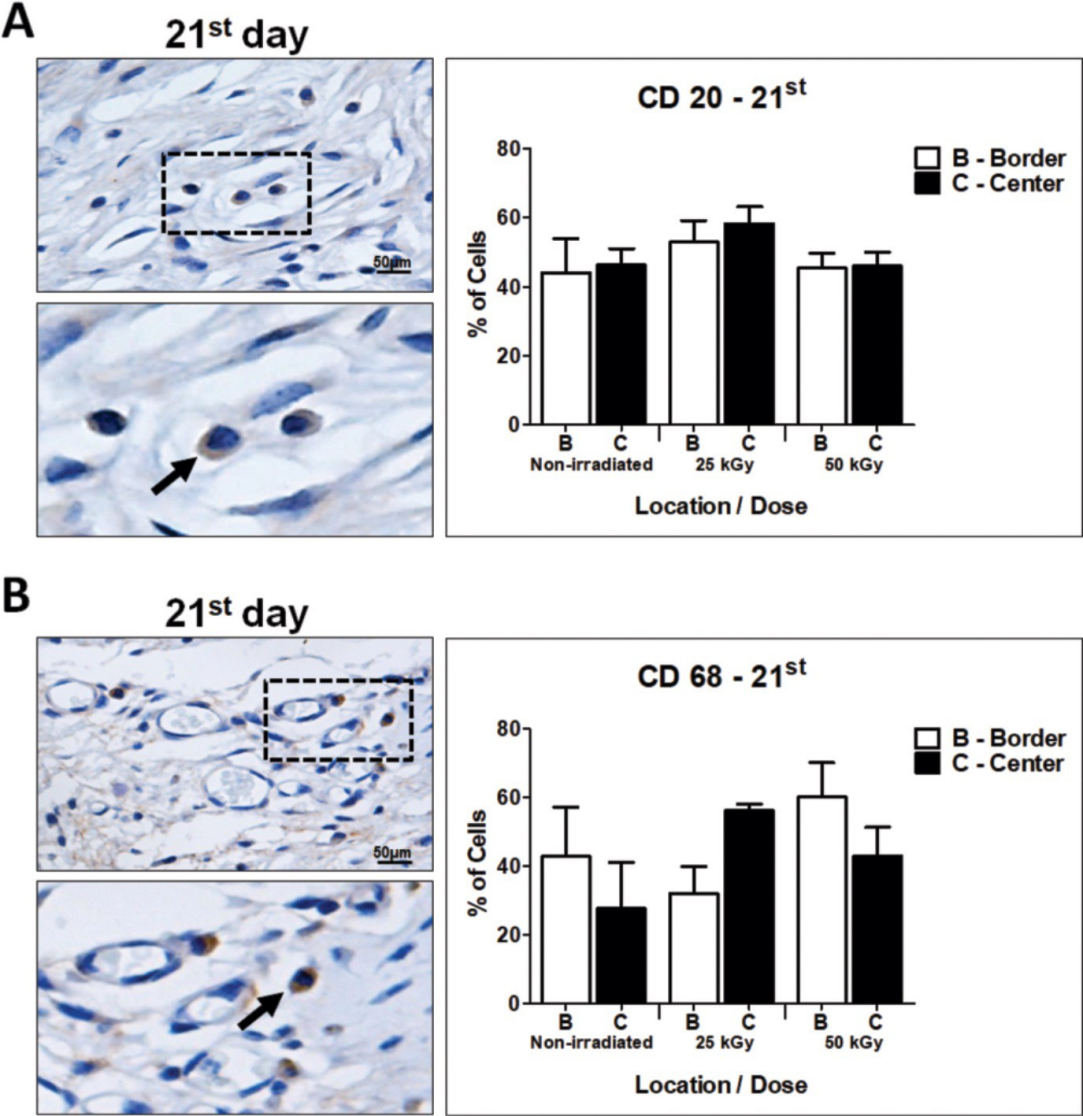
Distribution of B cells (CD20 +) and macrophages (CD68 +), 21 days after grafting. Tissue non-IHSG and IHSG 25 kGy and 50 kGy. Increased density of B-cell lymphocytes (CD20 +, arrow, A) and macrophages (CD68 +, arrow, B) did not show statistical significance, but a trend in mice from the IHSG 25 kGy group compared to the other groups. Original magnification: 400X (A, B); Lower figures: Zoom of the dashed region in A and B.

Fig 6 shows the distribution of B-cell lymphocytes (CD20+) and macrophages (CD68+) in the non-IHSG and IHSG 25 kGy and 50 kGy groups on the 21^st^ day after grafting. As expected, T-cells lymphocytes were absent, however an increased density of B-cell lymphocytes and macrophages (CD68+) were present around small vessels in the dermis of mouse in IHSG 25 kGy compared to non-IHSG and IHSG 50 kGy (Fig 6A and 6B).

## Discussion

The current study showed that the ionizing radiation at 25 kGy mediates inflammation and the repair stage of human skin graft adherence in murine model, thus emerging as a potential tool in healing cutaneous wounds. Our model was established using a stable human skin split-thickness graft with complete dermal and epidermal layers. Human skin allografts have been widely used as wound healing for a long time and are one of the most successful and widely used coverings for burn wound worldwide [19,21,22]. They can develop and organize the granulation tissue in the recipient wound. A human skin allograft becomes incorporated into the recipient tissue bed and will be covered by the recipient epidermal cells [23]. The Nude mice skin showed a high level of contraction in the implant location, notably during the first 21 days. In our model, half of the original size of the graft was observed after the IHSGs achieved a stable stage. The wound of 1.5 cm^2^ in the mice back was used to receive enough human graft, which developed the cutaneous wound healing after 21 days. The cutaneous wound evolution was the initial formation of a crust related to the dermal and epidermal surface area of the Nude mice. In 21 days, the IHSG site covered the crusty layer and a new keratinized and vascularized stratified epithelium was formed mimicking normal animal skin. Therefore, based on these results, we have used this experimental model to describe the inflammatory and remodeling stages of human skin allograft adherence in the Nude mice model. Skin stability was observed up to 90 days after the IHSGs implant, which demonstrates the effectiveness of our experimental model.

In the current study, Nude mice transplanted with IHSG 25 kGy resembled the beginning of an efficient healing, considering a significant decrease in dermo-epidermal necrosis, neutrophils, and lower loss of skin thickness, epithelization and vascularization, in addition to increased distribution of B-cell lymphocytes present around small vessels in the dermis 21 days post-IHSG 25 kGy grafting observed in this animal group. In fact, a B-cell response is expected in Nude mice, considering this animal model has deficient T-cell function, due a mutation which results in dysgenesis of the thymus. In addition, this model presents an enhanced macrophage activity as well the incapacity of rejecting skin allografts and xenografts [24]. Furthermore, our results show that after IHSG 25 kGy grafting small vessels were present around the dermis of mouse compared to non-IHSG and IHSG 50 kGy. It is worth emphasizing the importance of increasing B cells with 25 kGy IHSG when referring to grafts performed with other animal species or even human skin. In this case, the B cells can present antigens to T cells promoting priming and activation, and inducing tolerance. B cells can also modulate dendritic cells, CD8 and CD4 T cells through secretion of soluble factors such as transforming growth factor-β (TGF-β) or interleukin-10 (IL-10), inducing anergy or deletion of CD4 and CD8 cells directly [25,26]. Moreover, in this scenario, we observed a granulation tissue progressively invading the incision space and collagen fibers present in the margins of the incision, vertically oriented and not bridging the incision. Neovascularization was intense and the epidermis started to recover its normal thickness. It is noteworthy that the growth and proliferation of the keratinocytes layers in this healing stage can be related to the increase of fibroblasts and its mitogenic effect on the epithelial cells [27,28].

In addition, a complete repair of the mice wound occurred with IHSG 25 kGy after 21 days, characterized by the presence of fragments well-preserved human skin in the borders of wound, prominent granulation tissue in the organization by proliferated fibroblasts and deposition of fine collagen fibers in dermis. Since that inflammation is also responsible for the initiation of the repair process by activate fibroblasts, we infer that 25 kGy radiation dose may also explain the increased percentage of Col III fibers in our mice model. Similar findings were reported by Grinnell and Petroll [29], who suggested that the existence of porosity in the ECM facilitate the fibroblast migration between the collagen fibers, promoting change in dermal structure [20,30]. Other reports also support our results by emphasizing that radiation dose at 25 kGy promotes cell migration through the fibrillar structure of the dermis [31].

The crucial mechanisms for the normal skin ionizing radiation damage are degradation and subsequent remodeling of the connective tissue matrix by collagen synthesis. The structural alterations of collagen after irradiation in lower doses include modifications in its physic-chemical properties, indicating formation of a more “rigid” biopolymer structure, disruption of the parallel packing and reduction of the collagen fibers’ thickness [32]. According to our findings, the effects of 25 kGy radiation of human skin transplanted to the mice were observed after the first week post-ionizing radiation at the collagen fibers, showing decreased Col I at border and center of the human skin. We assign these changes of fibers Col I in human skin to the protein matrix modification which is known to occur as the first stage of the radiation damage development in the ECM. Nevertheless, the signs of the ECM modification visualized by immunofluorescence involved Col I fibers in well oriented bundles, resulting in a repair process that promote a better adherence of the skin graft.

However, one of the limitations of this study will be to evaluate the response of irradiated skin in knock out mice to clarify the possible mechanisms of mechanical activation that would facilitate the healing response of the skin tissue.

Considering the growing need for tissue biosafety and bioengineering of biomaterials in reconstructive plastic surgery, the possibility of expanding the scope of radio-sterilized grafts is a great challenge for the developed protocol and a good perspective for this area of research. We concluded that the ionizing radiation at 25 kGy mediates inflammation and the remodeling stage of human skin graft adherence in murine model, thus emerging as potential tool in healing cutaneous wounds.

## Supporting information

S1 Fig Representative image of the experimental protocol. The human skin from multi-donor sterilized by irradiation with 25kGy and 50kGy or non-irradiated were allografted in a Nude mice model. Skin samples of allografts were collected after 3, 7, 21 and 90 days to analysis.
(TIF)

S1 Data (PDF)

S2 Data (PDF)

S3 Data (PDF)

S4 Data (PDF)

S5 Data (PDF)

S6 Data (PDF)

## References

[1] Souto EB, Ribeiro AF, Ferreira MI, Teixeira MC, Shimojo AAM, Soriano JL, et al. New Nanotechnologies for the Treatment and Repair of Skin Burns Infections. Int J Mol Sci. 2020;21(2): 393. doi: 10.3390/ijms21020393 31936277 PMC7013843

[2] Tuğcu H, Zor F, Toygar M, Balandız H. Comparison of antemortem clinical diagnosis and postmortem findings in burn-related deaths. Ulus Travma Acil Cerrahi Derg. 2015 21(6): 491–5. doi: 10.5505/tjtes.2015.36604 27054641

[3] Finnerty CC, Jeschke MG, Branski LK, Barret JP, Dziewulski P, Herndon DN. Hypertrophic scarring: the greatest unmet challenge after burn injury. Lancet. 2016;388(10052): 1427–36. doi: 10.1016/S0140-6736(16)31406-4 27707499 PMC5380137

[4] Lobmann R, Ambrosch A, Schultz G, Waldmann K, Schiweck S, Lehnert H. Expression of matrix-metalloproteinases and their inhibitors in the wounds of diabetic and non-diabetic patients. Diabetologia. 2002;45(7): 1011–6. doi: 10.1007/s00125-002-0868-8 12136400

[5] Kellar RS, Diller RB, Tabor AJ, Dominguez DD, Audet RG, Bardsley TA, et al. Improved wound closure rates and mechanical properties resembling native skin in murine diabetic wounds treated with a tropoelastin and collagen wound healing device. J diabetes Clin Res. 2020;2(3): 86–99. doi: 10.33696/diabetes.1.024 33768213 PMC7990315

[6] Guillon C, Ferraro S, Clement S, Bouschbacher M, Sigaudo-Roussel D, Bonod C. Glycation by glyoxal leads to profound changes in the behavior of dermal fibroblasts. BMJ Open Diabetes Res Care. 2021;9(1): e002091. doi: 10.1136/bmjdrc-2020-002091 33903117 PMC8076933

[7] Baltzis D, Eleftheriadou I, Veves A. Pathogenesis and Treatment of Impaired Wound Healing in Diabetes Mellitus: New Insights. Adv Ther. 2014;31(8):817–36. doi: 10.1007/s12325-014-0140-x 25069580

[8] Tardáguila-García A, García-Morales E, García-Alamino JM, Álvaro-Afonso FJ, Molines-Barroso RJ, Lázaro-Martínez JL. Metalloproteinases in chronic and acute wounds: A systematic review and meta-analysis. Wound Repair Regen. 2019;27(4):415–20. doi: 10.1111/wrr.12717 30873727

[9] Grzela T, Krejner A, Litwiniuk M. Matrix metalloproteinases in the wound microenvironment: therapeutic perspectives. Chronic Wound Care Manag Res. 2016: 29–39. Available from: https://www.dovepress.com/matrix-metalloproteinases-in-the-wound-microenvironment-therapeutic-pe-peer-reviewed-fulltext-article-CWCMR.

[10] Chaganti P, Gordon I, Chao JH, Zehtabchi S. A systematic review of foam dressings for partial thickness burns. Am J Emerg Med. 2019;37(6):1184–90. doi: 10.1016/j.ajem.2019.04.014 31000315

[11] Shi C, Wang C, Liu H, Li Q, Li R, Zhang Y, et al. Selection of Appropriate Wound Dressing for Various Wounds.Front Bioeng Biotechnol. 2020;8: 182. doi: 10.3389/fbioe.2020.00182 32266224 PMC7096556

[12] Obeng MK, McCauley RL, Barnett JR, Heggers J., Sheridan K, Schutzler SS. Cadaveric allograft discards as a result of positive skin cultures. Burns 2001 27(3):267–71. doi: 10.1016/s0305-4179(00)00116-9 11311520

[13] Nita M, Pliszczyński J, Kowalewski C, Woźniak K, Eljaszewicz A, Moniuszko M, et al. New Treatment of Wound Healing With Allogenic Acellular Human Skin Graft: Preclinical Assessment and In Vitro Study. Transplant Proc. 2020;52(7): 2204–2207. doi: 10.1016/j.transproceed.2020.02.115 32340748

[14] Leon-villapalos J, Mohamed E, Dziewulski P. The use of human deceased donor skin allograft in burn care. Cell Tissue Bank. 2010;11(1): 99–104. doi: 10.1007/s10561-009-9152-1 20077178

[15] Franchini M, Zanini D, Bosinelli A, Fiorini S, Rizzi S, Aloja CD, et al. Evaluation of cryopreserved donor skin viability: the experience of the regional tissue bank of Verona. Blood Transfus. 2009;7(2): 100–5. doi: 10.2450/2008.0014-08 19503630 PMC2689063

[16] Herson MR, Ferreira MC. Split thickness skin allografts processed in glycerol >75%—Potential use as dermal regeneration matrix. Burns. 2007 Feb;33(1):S150. doi: 10.1007/s10561-018-9694-1 29696490

[17] Singh R, Singh D, Singh A. Radiation sterilization of tissue allografts: A review. World J Radiol. 2016;8(4): 355–70. doi: 10.4329/wjr.v8.i4.355 27158422 PMC4840193

[18] Dziedzic-Goclawska A, Kaminski A, Uhrynowska-Tyszkiewicz I, Stachowicz W. Irradiation as a safety procedure in tissue banking. 2005;6(3): 201–19. doi: 10.1007/s10561-005-0338-x 16151960

[19] Bourroul SC, Herson MR, Pino E, Matho MB. Sterilization of skin allografts by ionizing radiation. Cell Mol Biol (Noisy-le-grand). 2002;48(7): 803–7. 12619979

[20] Rooney P, Eagle M, Hogg P, Lomas R, Kearney J. Sterilisation of skin allograft with gamma irradiation. Burns. 2008;34(5): 664–73. doi: 10.1016/j.burns.2007.08.021 18226461

[21] Mahdavi-Mazdeh M, Nozary Heshmati B, Tavakoli SAH, Ayaz M, Azmoudeh Ardalan F, Momeni M. Human split-thickness skin allograft: Skin substitute in the treatment of burn. Int J Organ Transplant Med. 2013;4(3): 96–101. 25013660 PMC4089318

[22] Robb EC, Bechmann N, Plessinger RT, Boyce ST, Warden GD, Kagan RJ. Storage media and temperature maintain normal anatomy of cadaveric human skin for transplantation to full-thickness skin wounds. J Burn Care Rehabil. 2001;22(6): 393–6. doi: 10.1097/00004630-200111000-00008 11761390

[23] Mohd Yussof SJ, Halim AS, Mat Saad AZ, Jaafar H. Evaluation of the Biocompatibility of a Bilayer Chitosan Skin Regenerating Template, Human Skin Allograft, and Integra Implants in Rats. ISRN Mater Sci. 2011 Aug 28;2011:1–7. doi: 10.5402/2011/857483

[24] Pelleitier M, Montplaisir S. The nude mouse: a model of deficient T-cell function. Methods Achiev Exp Pathol. 1975;7: 149–66. 1105061

[25] Ashour HM, Seif TM. The role of B cells in the induction of peripheral T cell tolerance. J Leukoc Biol. 2007;82(5): 1033–9. doi: 10.1189/jlb.0507310 17656652

[26] Langier S, Galvani RG, Alves APG, Fidelis R, Nunes PHC, Silva MH, et al. Prolonged acceptance of skin grafts induced by B cells places regulatory T cells on the histopathology scene. Braz J Med Biol Res. 2012;45(10): 942–7. doi: 10.1590/s0100-879x2012007500089 22641417 PMC3854184

[27] El Ghalbzouri A, Lamme E, Ponec M. Crucial role of fibroblasts in regulating epidermal morphogenesis. Cell Tissue Res. 2002;310(2): 189–99. doi: 10.1007/s00441-002-0621-0 12397374

[28] Werner S. Keratinocyte Growth Factor: A Unique Player in Epithelial Repair Processes. Cytokine Growth Factor Rev. 1998;9(2): 153–65. doi: 10.1016/s1359-6101(98)00010-0 9754709

[29] Grinnell F, Petroll WM. Cell Motility and Mechanics in Three-Dimensional Collagen Matrices. Annu Rev Cell Dev Biol. 2010;26(1): 335–61. doi: 10.1146/annurev.cellbio.042308.113318 19575667

[30] Mrázová H, Koller J, Fujeríková G, Babál P. Structural changes of skin and amnion grafts for transplantation purposes following different doses of irradiation. Cell Tissue Bank. 2014;15(3): 429–33. doi: 10.1007/s10561-013-9407-8 24254127

[31] McQuestion M. Evidence-Based Skin Care Management in Radiation Therapy: Clinical Update. Semin Oncol Nurs. 2011;27(2): e1–17. doi: 10.1016/j.soncn.2011.02.009 21514477

[32] Tzaphlidou M, Kounadi E, Leontiou I, Matthopoulos DP, Glaros D. Influence of low doses of gamma -irradiation on mouse skin collagen fibrils. Int J Radiat Biol. 1997;71(1): 109–15. doi: 10.1080/095530097144481 9020970

