# Supplementary figures and images for: Sterilized human skin graft with a dose of 25 kGy provides a privileged immune and collagen microenvironment in the adhesion of Nude mice wounds
Source: PLoS One. 2022 Jan 27;17(1):e0262532. doi: 10.1371/journal.pone.0262532 (PMC8794154; doi:10.1371/journal.pone.0262532)

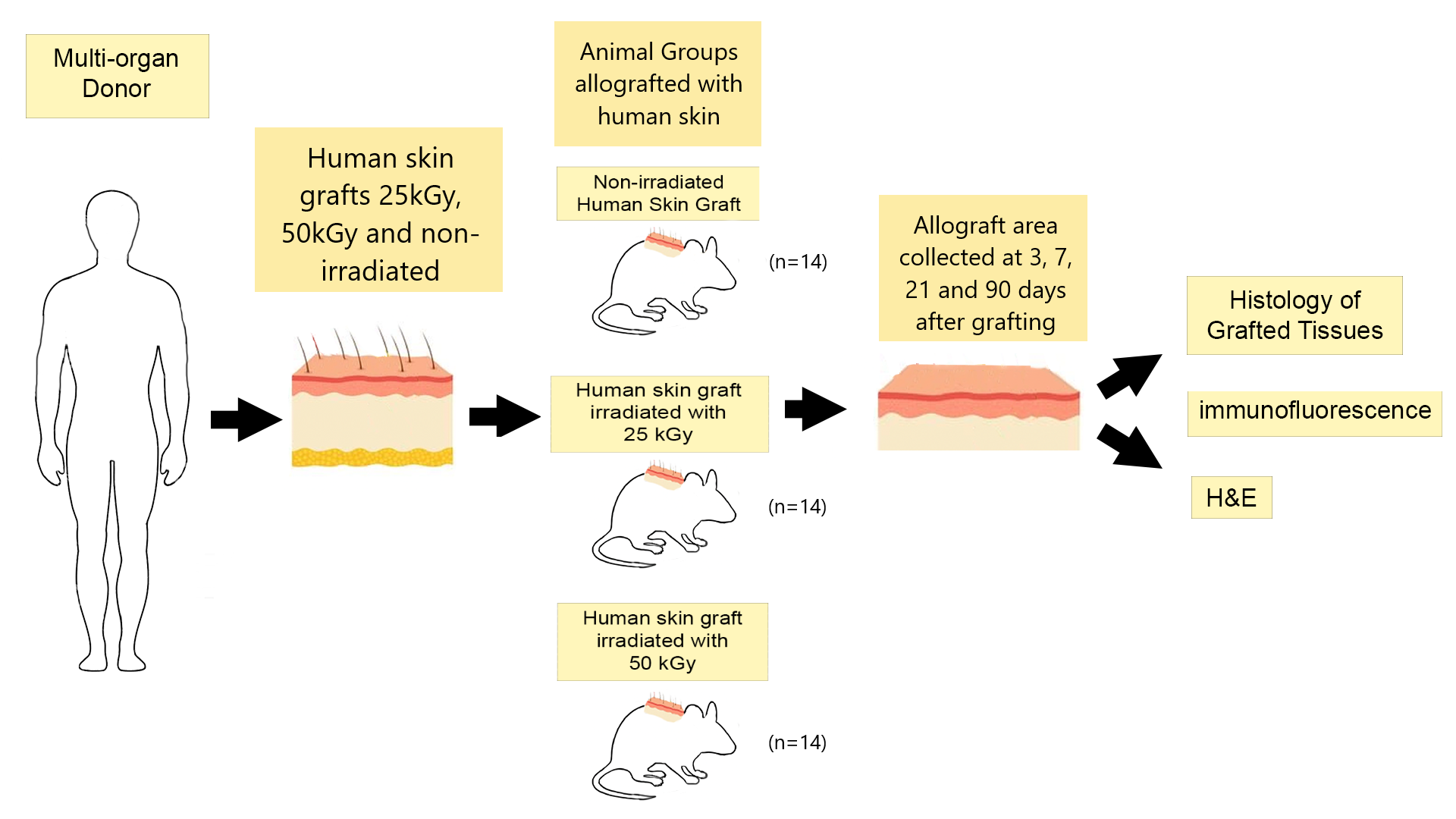

Supplement: S1 Fig — The human skin from multi-donor sterilized by irradiation with 25kGy and 50kGy or non-irradiated were allografted in a Nude mice model. Skin samples of allografts were collected after 3, 7, 21 and 90 days to analysis. (TIF) [file pone.0262532.s001.tif]
