# Supplementary material for: Sterilized human skin graft with a dose of 25 kGy provides a privileged immune and collagen microenvironment in the adhesion of Nude mice wounds
Source: PLoS One. 2022 Jan 27;17(1):e0262532. doi: 10.1371/journal.pone.0262532 (PMC8794154; doi:10.1371/journal.pone.0262532)
Supplement: S1 Data — (PDF) [file pone.0262532.s002.pdf]

N - Dermoep Necrosis	N - Neutroph	N - Skin Thickness
4	4	3
3	4	4
3	4	4
4	4	3
3	4	3
4	4	4
4	4	4
4	4	3
4	4	3
3	4	4
3	4	4
4	4	4

N - Epitheliz	N - Vasculariz	5kGy - Dermoep Necrosi	25kGy - Neutroph
0	1	2	2
1	1	2	2
1	1	2	3
1	1	2	2
0	2	2	3
2	2	3	2
1	1	3	2
1	1	2	2
1	1	3	3
0	1	2	2
0	2	2	2
0	2	2	2

25kGy - Skin Thickness	25kGy - Epitheliz	25kGy - Vasculariz	0kGy - Dermoep Necrosi
2	3	3	3
3	3	2	3
3	2	2	3
2	2	3	2
2	2	2	3
3	3	3	3
3	3	3	2
2	2	2	2
2	3	2	3
2	3	2	3
3	3	3	3
3	3	2	3

50kGy - Neutroph	50kGy - Skin Thickness	50kGy - Epitheliz	50kGy - Vasculariz
3	4	2	2
3	4	2	2
3	3	2	2
3	3	2	2
3	3	2	2
2	2	1	1
3	3	1	1
3	3	2	1
2	2	2	1
3	3	3	2
3	3	1	2
3	3	2	2
