# Supplementary material for: Sterilized human skin graft with a dose of 25 kGy provides a privileged immune and collagen microenvironment in the adhesion of Nude mice wounds
Source: PLoS One. 2022 Jan 27;17(1):e0262532. doi: 10.1371/journal.pone.0262532 (PMC8794154; doi:10.1371/journal.pone.0262532)
Supplement: S2 Data — (PDF) [file pone.0262532.s003.pdf]

Granulation tissue	Fibroblasts	Collagen
2	1	2
2	2	1
1	2	1
2	1	2
2	1	2
1	2	1
2	2	1
2	1	1
1	2	2
2	1	2
2	2	2
1	1	1

Reepithelization	25 - Granulation tissue	25 - Fibroblasts	25 - Collagen
2	4	3	4
2	4	4	4
1	4	4	3
2	4	3	3
1	3	4	4
1	3	4	4
2	4	3	4
2	4	3	3
1	3	4	3
1	3	4	4
2	2	4	4
1	3	3	4

25 - Reepithelization	50 - Granulation tissue	50 - Fibroblasts	50 - Collagen
3	2	2	3
3	2	2	2
3	2	2	2
3	2	2	2
3	3	2	3
3	3	3	2
3	2	3	2
3	2	2	2
2	2	2	2
2	2	1	3
2	2	2	2
2	2	1	3

50 - Reepithelization
1
1
2
2
1
1
2
1
2
1
1
2
