# Supplementary material for: Sterilized human skin graft with a dose of 25 kGy provides a privileged immune and collagen microenvironment in the adhesion of Nude mice wounds
Source: PLoS One. 2022 Jan 27;17(1):e0262532. doi: 10.1371/journal.pone.0262532 (PMC8794154; doi:10.1371/journal.pone.0262532)
Supplement: S3 Data — (PDF) [file pone.0262532.s004.pdf]

Non-irradiated	25 kGy	50 kGy
22,55623	5,385549	39,34563
22,11112	8,97486	18,63082
42,74144	1,793087	3,167208
13,35734	0,7817653	17,63362
42,33374	9,784805	21,55005
72,09467	19,84418	30,2867
41,84771	6,141109	42,69611
48,70746	11,81633	41,08324
30,98278	2,265755	6,605639
20,08214	5,30633	6,112011
15,33808	3,412774	3,687255
50,16077	1,376754	18,76962
17,71271	5,282082	0,3419808
50,6863	5,297817	3,976931
45,64645	6,757199	4,548731
46,69493	6,221564	13,94287
35,47562	12,83464	0,7232097
27,20463		
	9,612746	17,2618
56,67041	3,555321	18,80098
74,41064	8,634472	12,12513
55,44253	3,16836	
67,41852		8,313283
72,29309	7,091429	5,945895
20,28234	2,309197	17,7713
	8,192463	16,30799
60,93792	1,903719	11,66996
62,52923		
60,43944		
33,51604		
