# Supplementary material for: Sterilized human skin graft with a dose of 25 kGy provides a privileged immune and collagen microenvironment in the adhesion of Nude mice wounds
Source: PLoS One. 2022 Jan 27;17(1):e0262532. doi: 10.1371/journal.pone.0262532 (PMC8794154; doi:10.1371/journal.pone.0262532)
Supplement: S4 Data — (PDF) [file pone.0262532.s005.pdf]

Non-irradiat	25 kGy	50 kGy
3,90399	5,7028	3,25583
16,7524	7,27977	1,41157
2,49472	13,3735	1,36891
11,649	24,0017	7,05935
8,33751	25,6031	5,36595
25,4446	13,502	2,26677
7,68378	26,1084	12,8965
2,77392	21,8549	15,6799
5,81071	14,1559	34,3212
9,67567	27,5813	21,8313
4,94791	15,1015	7,93261
	21,7669	6,87252
6,68259		
43,6075	5,96172	2,0047
47,6117	7,08587	5,8244
24,7688	4,20639	4,79665
27,6883	6,30412	13,7845
10,8638	4,47879	
		25,9106
4,09128	22,938	21,4252
19,0541	27,148	28,8545
5,48122	7,21351	42,1212
17,0074	2,9672	12,906
	7,58514	42,5137
20,4346	2,87395	
22,8429		3,62903
6,46482	28,4358	2,07135
7,04925	25,4783	0,61191
	22,3232	0,95131
0,57133	13,3487	
0,73022		28,4394
0,33556	13,7716	23,51
5,04883	8,22245	11,6435
	15,4479	23,3698
76,999	16,5421	
43,327		3,84511
90,0296	1,89075	3,69308
33,8487	3,36701	4,53472
	4,84178	16,5262
20,7764	5,82349	
12,7348		16,9544
17,9023	21,0151	25,2945
8,49722	25,827	15,9427
	6,13727	40,5444
	10,451	
