# Supplementary material for: Sterilized human skin graft with a dose of 25 kGy provides a privileged immune and collagen microenvironment in the adhesion of Nude mice wounds
Source: PLoS One. 2022 Jan 27;17(1):e0262532. doi: 10.1371/journal.pone.0262532 (PMC8794154; doi:10.1371/journal.pone.0262532)
Supplement: S5 Data — (PDF) [file pone.0262532.s006.pdf]

Non-irradiated	25 kGy	50 kGy
15,48828	29,80529	39,7053
18,2756	36,42365	25,27982
20,47077	35,88796	35,6799
24,02162	35,23127	35,38015
20,3554	31,03048	20,94348
19,87396	26,21803	24,98628
24,71039	55,32146	38,00676
31,48548	59,54332	36,23944
42,55445	32,45681	35,65112
28,46566	41,19056	37,13384
32,99422	46,73386	36,93803
52,3683	34,71803	17,67131
18,42505	26,75211	21,1205
14,7421	27,66964	33,76981
19,77201	23,12915	29,2864
4,233675	33,15581	41,58514
8,875713	29,95019	18,40549
	39,23693	17,30738
18,60781		
18,43048	29,09558	16,55109
12,67433	15,59304	15,67745
20,61826	30,63335	42,66615
10,08393	22,93077	36,15023
6,724784	46,43871	12,26262
	37,59726	31,47122
29,59297		
37,46125	40,21651	15,03215
28,15398	30,16271	14,09539
25,66854	16,32603	27,39872
24,42107	49,36783	24,8263
	30,24125	33,92755
	44,96389	13,95753
