# Supplementary material for: Sterilized human skin graft with a dose of 25 kGy provides a privileged immune and collagen microenvironment in the adhesion of Nude mice wounds
Source: PLoS One. 2022 Jan 27;17(1):e0262532. doi: 10.1371/journal.pone.0262532 (PMC8794154; doi:10.1371/journal.pone.0262532)
Supplement: S6 Data — (PDF) [file pone.0262532.s007.pdf]

| N - Macrophages | N- Revascularization | 25 - Macrophages |
|---|---|---|
| 2 | 2 | 3 |
| 2 | 2 | 3 |
| 2 | 2 | 3 |
| 2 | 2 | 2 |
| 2 | 2 | 2 |
| 1 | 2 | 3 |
| 1 | 2 | 3 |
| 1 | 2 | 2 |
| 2 | 2 | 2 |
| 2 | 2 | 3 |
| 2 | 2 | 3 |
| 2 | 2 | 3 |

| 25 - Revascularization | 50 - Macrophages | 50 - Revascularization |
|---|---|---|
| 4 | 2 | 2 |
| 3 | 2 | 2 |
| 3 | 2 | 2 |
| 4 | 2 | 1 |
| 4 | 2 | 1 |
| 3 | 2 | 2 |
| 3 | 2 | 2 |
| 4 | 2 | 1 |
| 4 | 2 | 1 |
| 4 | 2 | 2 |
| 3 | 2 | 2 |
| 3 | 2 | 2 |
